# Implications of vaccine-delivery policy for dermatologic care: Lessons from recent US recommendations

**DOI:** 10.1016/j.jdin.2026.08.007

**Published:** 2026-08-29

**Authors:** Umayr R. Shaikh, Afrin Mirza, Nabiha Iqbal, Alina Galaria, Harold William Higgins, Jane M. Grant-Kels

**Affiliations:** a Georgetown University School of Medicine, Washington, DC; b Sidney Kimmel Medical College at Thomas Jefferson University, Philadelphia, Pennsylvania; c Department of Dermatology, University of Pennsylvania, Philadelphia, Pennsylvania; d Department of Dermatology, University of Connecticut Farmington, Connecticut; e Department of Dermatology, University of Florida Health, Gainesville, Florida

**Keywords:** dermatology, health care access, immunosuppression, public health, vaccination, vaccine policy

*To the Editor:*

On August 10, 2026, a US executive order established “Gold Standard Childhood Vaccine Recommendations,” retaining human papillomavirus and varicella vaccination among immunizations recommended for all children while reassigning others to high-risk or shared clinical decision-making categories.^1^ Two additional provisions address vaccine delivery. First, measles, mumps, and rubella should be administered as three single-disease shots “once such products are domestically available.” Second, “to the maximum extent feasible,” all childhood immunization should be given at separate medical visits.^1^ Although implementation remains uncertain, the proposal raises a broader question relevant to dermatologists internationally: how can vaccine-delivery policy affect access to timely dermatologic treatment?

Dermatologists increasingly prescribe immunomodulatory therapies for psoriasis, atopic dermatitis, autoimmune blistering diseases, and other inflammatory dermatoses, making immunization review an important component of treatment initiation.^2^ Live vaccines generally should be completed before immunosuppression begins.^2^ Vaccination logistics, not simply which vaccines are recommended, can therefore influence when systemic treatment can safely begin. An incompletely immunized adolescent may currently receive multiple indicated vaccines during one encounter. Policies discouraging coadministration could instead prolong this process through repeated appointments and necessary dosing intervals.

Although prompted by a US policy proposal, this issue is not uniquely American. Immunization schedules, vaccine availability, and health care delivery models differ substantially across countries; yet minimizing missed opportunities for vaccination is a shared public health priority.^3^ Additional encounters introduce transportation costs, caregiver work-absence, scheduling difficulties, and other barriers. These burdens may be particularly consequential in health care systems with limited primary care capacity, fragmented specialty access, geographic barriers, or constrained vaccine availability. Regardless of the national vaccination schedule, increasing the number of encounters required for immunization may compound barriers to specialty treatment. For dermatology patients awaiting immunomodulatory therapy, inefficient vaccine delivery may therefore translate into delayed disease control.

Evidence from the US illustrates the importance of delivery structure. In a nationally representative sample, children receiving at least one combination vaccine completed the recommended series by 24 months more frequently than those receiving only single-antigen vaccines (69% vs 50%).^4^ After adjustment for individual, household, and provider characteristics, they were 2.5 times as likely to complete the series and 2.2 times as likely to be vaccinated on time.^4^ Although not directly evaluating separate-visit policies, these data demonstrate that vaccine delivery can influence completion and timeliness.

Dermatologists can help mitigate these barriers by identifying immunization gaps early and coordinating vaccination before immunosuppression. Where health care systems permit, dermatology clinics may also administer vaccines directly. Prior dermatology-based vaccination initiatives demonstrate that specialty clinics can function as vaccination touchpoints, an approach that may be particularly valuable for patients with fragmented primary care access.^5^

The clinical effect of the US executive order remains uncertain, but its broader lesson extends beyond US borders. Vaccine policy should be evaluated not only by which immunization are recommended, but by the treatment burden created by how they are delivered. Dermatologists should support flexibility in coadministration, early identification of immunization gaps, expedited preimmunosuppression vaccination pathways, and strong specialty–primary care coordination so that evolving vaccination policies do not inadvertently delay dermatologic care.

## Conflicts of interest

None disclosed.
